# Reminiscences

**Published:** 1886-04

**Authors:** 


### REMINISCENCES OF CANNING.

Dr. N. S. Davis speaks of that part of science which has found final resting place in the Text-books—the accepted—the known, as ''canned science,'' (put up in its own juice, as it were.) Pretty good.

